# Monitoring of Volatile Organic Compounds in Strawberry Genotypes over the Harvest Period

**DOI:** 10.3390/plants12091881

**Published:** 2023-05-04

**Authors:** Kondylia Passa, Carmen Simal, Evangelos Tsormpatsidis, Vasileios Papasotiropoulos, Fotini N. Lamari

**Affiliations:** 1 Laboratory of Agricultural Genetics & Plant Breeding, Department of Agriculture, University of Patras, 30200 Messolonghi, Greece; kondyliapassa@gmail.com; 2 Laboratory of Pharmacognosy & Chemistry of Natural Products, Department of Pharmacy, University of Patras, 26504 Patras, Greece; carmen.simal@gmail.com; 3 Berryplasma World Ltd., 27052 Varda, Greece; e.tsormpatsidis@berryplasma.gr

**Keywords:** *Fragaria* × *ananassa*, VOCs, key aroma components, gas chromatography–mass spectrometry, harvest time, breeding

## Abstract

Volatile Organic Compounds (VOCs) over the harvest period have been assessed in twenty-five strawberry genotypes cultivated in western Greece. Using liquid–liquid extraction and gas chromatography–mass spectrometry (GC–-MS), twenty-eight volatiles were monitored at early (T1) and mid-harvest (T3) time points to investigate the effect of the genotype and harvest time on strawberry volatilome. A quantitative impact of both harvest date and genotype on VOCs associated with aroma was demonstrated, with the most significant VOCs being terpenes, esters, and acids, followed by lactones and furanones. Harvest date was crucial for terpenoid and phenylpropanoid content, and important for esters, short-chain acids, and lactones. Six out of the twenty-five genotypes (four commercial varieties, including ‘Rociera’, ‘Victory’, ‘Leyre’, and ‘Inspire’, and two advanced selection genotypes (G2 and G8) were evaluated at two additional time points, covering the entire harvest season. The volatile levels were higher in fruits harvested at early stages (T1–T2) for most of the genotypes examined. The G2 genotype turned out to have a less ample but more stable volatile profile throughout harvesting, while ‘Victory’, ‘Leyre’, and ‘Inspire’ exhibited less abrupt changes than ‘Rociera’. This study demonstrates that the determination of VOCs provides significant information regarding the differences in strawberry genotypes related to aroma and enables the selection of genotypes based on specific VOCs content and/or volatile stability over the harvest period. Furthermore, this study pinpoints that growers could opt for optimal harvest dates based on the genotypes and the VOC content.

## 1. Introduction

The cultivated strawberry (*Fragaria* × *ananassa* (Duchesne ex Weston) Duchesne ex Rozier) is an herbaceous hetero-octaploid plant of the Rosaceae family, which emerged in France in the 18th century from an accidental cross between the North American octoploid *Fragaria virginiana* and the South American octoploid *Fragaria chiloensis* [1]. It is one of the most economically significant berry fruit worldwide, with a global production of about 9 million tons in 2020, of which 1,664,506 is being produced in Europe and 84,220 in Greece [2]. Strawberries enjoy great popularity among consumers, not only for their high nutritional value but also for their distinct aroma and flavor. The perception of this pleasant aroma is attributed to the emission of the volatile organic compounds (VOCs) they contain, although they represent only 0.01% of the fresh fruit mass [3].

Modern breeding programs aim to expand strawberry cultivation by developing varieties with improved traits in terms of disease and pest resistance, drought, heat and cold tolerance, flowering habit, yield, and adaptability to mechanical harvest [4]. Furthermore, the development of cultivars with the ability to adapt to regional cultivation conditions, possessing also high fruit quality in terms of organoleptic characteristics and increased aroma volatiles is also of great importance [5]. To date, nearly a thousand volatile compounds have been identified in strawberry fruit, including a plethora of chemical families and structures from secondary metabolites [6,7,8]. Tressl et al. [9] detected more than 200 volatile compounds in the *F.* × *ananassa* cultivar cv. ‘Revata’, whereas Cannon et al. [10] identified 567 volatiles in Ciflorette strawberries (*F.* × *ananassa* cv. ‘Ciflorette’). Those volatiles are classified mainly into alcohols, ketones, aldehydes, and aromatic compounds; however, the major contributors to strawberry aroma are not more than twenty metabolites, primarily identified as esters, acids, furanones, terpenes, and sulfur compounds [11]. A number of studies have demonstrated the following as primary flavor and aroma components: furanones, namely 2,5-dimethyl-4-methoxy-3(2*H*)-furanone (DMMF or mesifurane) and 2,5-dimethyl-4-hydroxy-3(2*H*)-furanone (DMHF or furaneol); esters, namely ethyl butanoate, methyl butanoate, methyl 2-methylpropanoate, ethyl hexanoate, and methyl hexanoate; terpenes, namely linalool and nerolidol; lactone, namely *γ*-decalactone; ketones, namely 2,3-butanedione and 2-heptanone; aldehyde, namely (*Z*)-3-hexenal; acids, namely butanoic acid and hexanoic acid [10,11,12,13,14]. Mesifurane has a toffee-like, sugary, and sweet odor, and furaneol has a candy and caramel one [10]. Ethyl butanoate gives a sweet and fruity note [10], methyl hexanoate a fresh and sweet flavor impact [15], and ethyl hexanoate is reminiscent of the apple aroma like *(Z*)-3-hexenal [10,15]. A peachy odor emerges due to *γ*-decalactone, while linalool gives a floral aromatic perception [10]. 2,3-Butanedione accounts for the sweet odor, while butanoic and hexanoic acids for a cheesy one [10].

In recent decades, numerous investigations have focused on the factors that may influence the levels of volatile compounds in strawberries and particularly on the genotype [16,17], the fruit’s maturity stage [18], the cultivation practices and the environment [16,19,20], and finally postharvest treatment [21]. It has been verified that the volatile profile varies among different strawberry cultivars [11], with the most diversified compounds being furanones (mesifurane and furaneol), lactones, acids, and esters. Some of them have been found to have high esters or terpenes content, such as linalool, while others have low esters levels and even a complete absence of furanones [22]. Aubert et al. [18] reported superior levels of volatiles in the ripe fruit of ‘Gariguette’ strawberries, in contrast to the lower levels observed in strawberries harvested when 75% red. Specifically, the levels of mesifurane, furaneol, ethyl butanoate, methyl hexanoate, linalool, nerolidol, and *γ*-dodecalactone were 2–12 times higher in fully ripe strawberries than those harvested at earlier maturity stages. The effect of analytical techniques is also great; for instance, the introduction of volatiles by solid-phase microextraction (SPME) and/or headspace to gas chromatography (GC) shows the esters in a much higher abundance, whereas the liquid–liquid extraction procedures downsize their contribution and increase that of acids and furanones [6,8].

The volatile composition is crucial for the fruit’s aroma and flavor; however, it is a complex phenomenon that depends on the genotype, the environmental conditions, the maturity level, and the production stage of the plants (early—middle—late harvesting) [23]. Therefore, the development of genotypes with an optimum and stable chemical composition throughout the harvest period is an important breeding target. Only a few studies have examined the volatile composition of strawberry fruit across the harvest season; Watson et al. [24], pointed out that the harvest date plays a crucial role in the presence and quantity of a range of volatile compounds in strawberry fruit, although a consistent expression pattern was not observed. Pelayo-Zaldivar et al. [25] demonstrated that the furaneol content increased in the middle of the harvest season and not at the beginning and that the methyl/ethyl esters ratio was also affected by the harvest date. The results of Jouquand et al. [26] showed that the levels of some volatile chemical families were affected by the harvest period, especially esters, terpenes, and lactones. In contrast, Schwieterman et al. [27] demonstrated that a later harvest leads to a reduced volatile content, which eventually erodes the overall liking of the fruit. Moreover, strawberry’s volatilome has been shown to be altered by postharvest treatments such as antimicrobials, freezing and thawing, and storage temperatures [22,28,29]. We have previously [30] adopted a liquid–liquid extraction technique for the analysis of volatiles in six strawberry genotypes and have demonstrated that the interaction between the genotype and harvest time had a significant impact (*p* < 0.001) on all tested quality attributes and flavor gene expression levels, with the exception of the volatile anhydrides, fatty acids, aromatics, and phenylpropanoids (all of which were greatly affected by the harvest time); in addition, lactones, furaneol, and FaEGS2 were affected solely by the genotype. However, the harvest time points in that study were in March and April and did not cover the early harvest period.

In this study, we present the screening of 25 strawberry genotypes cultivated in Western Greece concerning the abundance and chemical diversity of their volatilome at two different harvest time points: (a) at the beginning of the fruiting period (T1: 22 November 2021–31 December 2021) and (b) in the middle of the harvesting period (T3: 1 February 2022–15 March 2022). Our aim was to investigate the impact of the harvest date and the genotype on volatiles; this could be useful to breeders for the development of new cultivars with improved traits and to producers to satisfy the consumers liking demands. The evaluated genotypes comprised eight commercial varieties, grown in the region, as controls; ‘Rociera’, ‘Victory’, ‘Fortuna’, ‘Fronteras’, ‘Leyre’, ‘Inspire’, ‘Savana’, and ‘Plared-1075’ (G18–G25), three new varieties developed at Berryplasma World Ltd. (‘G13’, ‘G14’, and ‘G15’), five genotypes from their collection (G10, G11, G12, G16, and G17), and nine advanced selections (G1–G9). In addition, 6 genotypes out of the initial 25 were further studied at two more time points for an in-depth understanding of volatile composition changes over the harvesting season (T1: 22 November 2021–31 December 2021, T2: 1 January 2022–31 January 2022, T3: 1 February 2022–15 March 2022, and T4: 1 April 2022–30 April 2022). Those genotypes comprised the four commercial varieties (‘Rociera’, ‘Victory’, ‘Leyre’, and ‘Inspire’) and two advanced selections (G2 and G8).

## 2. Results and Discussion

### 2.1. Determination of Volatile Organic Compounds

A total of 25 strawberry genotypes (G1–G25, Table S1) were initially evaluated at the beginning of the fruiting period (T1) and the middle of the harvest period (T3). The composition and profiles of volatile organic compounds (VOCs) of all cultivars were analyzed via gas chromatography coupled with mass spectrometry (GC-MS). A complex blend of more than a hundred volatile compounds was identified in the LLE extracts and quantified in terms of chromatographic peak areas. Those with a relative abundance of at least 0.1% at one measurement (58 VOCs, Table S2) were monitored to study variations among cultivars at the harvest time points selected. Table 1 shows the most aroma-impactful and abundant volatile constituents (28 VOCs) in the examined samples at T1 and T3, 12 of which have been previously described by Ulrich et al. [6] as the most frequent volatiles in strawberries. Considering the amplitude of the range of the peak areas presented by most of the compounds, it can be concluded that there are no volatile emission patterns but rather a great diversity in the content levels across genotypes. Upon the results obtained from the statistical analysis though, a significant effect (*p* ≤ 0.05) was noted in few of the key aroma components previously mentioned (Table 1). These findings are consistent with prior studies that reported a wide spectrum of volatile phenotypes among cultivars, thus highlighting the effect of the genotype [16,22,31,32].

Based on the average %content values obtained from the detailed analysis of all genotypes at T1 and T3 (Table S3), VOCs were primarily classified as esters 4.1% (T1)/4.7% (T3), terpenes 6.5% (T1)/6.1% (T3), acids (short-chain acids 16.5% (T1)/16.3% (T3); fatty acids 39.1% (T1)/26.6% (T3)), phenylpropanoids 39.1% (T1)/26.6% (T3), lactones 6.7% (T1)/6.4% (T3), furanones 4.1% (T1)/9.7% (T3), anhydrides 1.9% (T1)/0.4% (T3), and other aromatic compounds 1.0% (T1)/2.9% (T3). It is also worth mentioning that detection of esters and terpenes (4.1 and 6.1%, respectively), even at low concentrations, represents a significant improvement compared to the previous study conducted in our laboratory, where the values observed were much lower for both chemical families (0.2 and 0.7%, respectively) [30]. This fact might be attributed to the possible pH changes to which the samples were subjected during their processing. In contrast, in this present work, salt (NaCl) was initially mixed with the fruit tissue (1:1, m/m) before homogenization to prevent any enzymatic activity [33] and to enhance the dissociation of VOCs [34], which were extracted afterward with the organic solvent (ethyl acetate, EtOAc). Furthermore, to minimize the presence of acids and anhydrides eluted from the HP-5MS column, basic–acidic treatments were performed during VOC extraction. Thus, in an attempt to separate and isolate the acidic compounds from the neutral–basic fraction, the homogenized samples were initially treated with NaHCO_3_ (0.5 M). The combined aqueous solutions were then adjusted to pH 2.0 with HCl (2 M) and extracted with EtOAc to release the acid fraction [35]. Both the acid and the neutral-base fractions were concentrated and analyzed by GC-MS. Unfortunately, a proper separation was not achieved, and the acids were detected in both extracts, so the protocol had to be rejected. Eventually, the volatiles were isolated by direct solvent extraction (see further details in Section 3.4).

According to the experimental data, the *trans*-(*E*)-cinnamic acid dominated in all genotypes, with an average %content of 38.2% at T1 and 24.9% at T3, whereas hexanoic acid ranked second at 12.5% (T1)/11.5% (T3). They were followed by a series of lactones and furanones, such as *γ*-decalactone 3.5% (T1)/3.9 (T3), *γ*-butyrolactone 2.8% (T1)/2.5% (T3), mesifurane 2.1% (T1)/3.4% (T3), and furaneol 2.0% (T1)/6.4% (T3), whose presence was quite minor, andthey are well known for their significant contribution to the intense aroma of strawberries [10,11,12,13,14,36]. Other important and highly frequent in the fruit volatile aroma compounds [6] were detected at lower %contents around 1–2%; e.g., butanoic 2.0% (T1)/1.4% (T3), 2-methylbutanoic acids 1.3% (T1)/2.6% (T3), esters, such as ethyl hexanoate 1.2% (T1)/1.4% (T3) and ethyl butanoate 1.1% (T1)/1.1% (T3), and terpenes, which were mainly dominated by *trans*-(*E*)-nerolidol 1.4% (T1)/0.9% (T3) and linalool 1.1% (T1)/1.1% (T3).

At the genotype level, G11 (9.6%) and G15 (10.8%) have a higher ester %content at T3 than the control varieties (with an average % content of 5.7%), while concerning terpenes, G13 stands out, exceeding 13% in both T1 and T3. Short chain acids were greater in ‘Rociera’ and ‘Plared-1075’ at T3 (47.1% and 46.1%, respectively), while for furanones, G8 and G9 had higher % content of mesifurane (7.5 and 9.9%) and furaneol (12.2 and 8.3%) than the control genotypes at T1. In addition, G8 showed at T3 the maximum furaneol %content observed, reaching up to 20.7%. With respect to lactones, the control varieties exhibited the highest percentage ratings; ‘Fortuna’, ‘Leyre’, ‘Savana’, and ‘Plared-1075’ had values above 13% in both T1 and T3, followed by G11, which exceeded 10% (Table S3).

### 2.2. Effect of Harvest Time on Strawberry Volatiles

**Figure 1:**
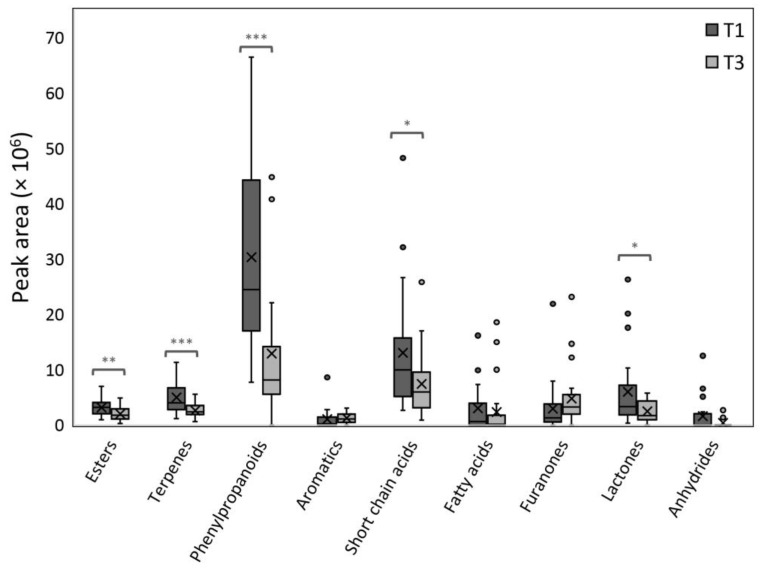
Box-plot graphical representation of the abundances in terms of chromatographic peak area (y-axis) of volatile main categories (x-axis) at harvest times T1 and T3 for the 25 genotypes examined. Asterisks indicate a statistically significant change in values (* 0.05 ≥ *p* > 0.01; ** 0.01 ≥ *p* > 0.001; *** *p* ≤ 0.001). Dots represent the outliers observed.

Figure 1 shows the concentration of the volatile categories of the 25 strawberry genotypes over the two time points (T1–T3). The harvest date played an essential role in the terpenoid and phenylpropanoid content (*p* ≤ 0.001) and, to a lesser extent, in esters, short-chain acids, and lactones (*p* ≤ 0.05) (Table 2). Jouquand and Chandler [26] have previously reported that the levels of total esters and total terpenes of all examined genotypes were also affected throughout the harvest season. In our study, the total volatile content among genotypes was significantly higher in T1 than in T3 for most categories, except for some outliers detected in specific genotypes. Fatty acids and anhydrides did not exhibit statistically significant variations from T1 to T3, nor did aromatic compounds and furanones, although, in the latter chemical classes, an increase from T1 to T3 was observed. In the list of VOCs, only a few ingredients proved to be highly harvest time-dependent, such as ethyl 2-methylbutanoate or *trans*-(*Ε*)-nerolidol, as presented in Table 2 (Harvest T1/T3). Other volatiles such as butanoic acid, furaneol, linalool, and *γ*-decalactone showed to be influenced by the harvest and the genotype, as earlier mentioned (Table 1), whereas mesifurane and *trans*-linalool oxide, were influenced predominantly by the genotype, an observation which could be important for breeding [31].

Additional time points, T2 and T4, were further examined to get insights into the changes in the volatiles throughout the whole harvest season (Table S4). For this purpose, 6 cultivars out of the 25 initially studied were selected based on agronomic criteria and their volatile content. Those included the commercial varieties of ‘Rociera’, ‘Victory’, ‘Leyre’, and ‘Inspire’, which showed the highest concentrations of most VOC species at T1 and T3, and two from Berryplama World Ltd. collection (G2 and G8). When the selected genotypes were evaluated at all time points, fewer volatile constituents were significantly affected by the harvest time, which might be due to the reduction of the genotypes studied and the shorter intervals among the time points (Table 2).

**Figure 2:**
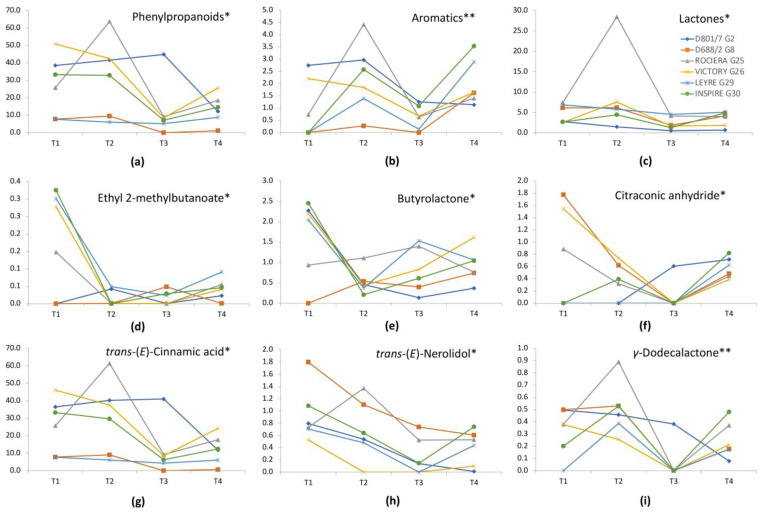
Evolution of the content levels of those constituents that presented statistically significant changes (* 0.05 ≥ *p* > 0.01; ** 0.01 ≥ *p* > 0.001) in the six selected genotypes over harvesting at the four time-points T1–T4 (chromatographic peak area (×10^6^) vs. harvest time point): (**a**) phenylpropanoids *, (**b**) aromatic compounds **, (**c**) lactones *, (**d**) ethyl 2-methylbutanoate *, (**e**) butyrolactone *, (**f**) citraconic anhydride *, (**g**) *trans*-(*E*)-cinnamic acid *, (**h**) *trans-*(*Ε*)-nerolidol *, and (**i**) *γ*-dodecalactone **.

Esters reached their maximum concentration in three out of the six genotypes, ‘Rociera’, ‘Inspire’, and ‘Leyre’, when harvested at T2, while the Berryplasma genotypes G2 and G8 showed the highest ester content in T1 and ‘Victory’ in T4. Our results agree with earlier observations that the ratio of methyl/ethyl esters was dependent on both genotype and harvest date [25]. In our study, ethyl 2-methylbutanoate, frequently mentioned as major strawberry aroma contributor, exhibited statistically significant change over harvesting period, with the maximum values recorded at T1 for all the commercial varieties (Figure 2). The genotype G8 presented the highest total content of terpenes in all time points (Figure S1); among them, *trans*-(*Ε*)-nerolidol showed a statistically significant decrease from T1 to T4 (Figure 2). The control varieties ‘Rociera’ and ‘Inspire’, on the other hand, showed the same trend as previously observed for the esters, showing the most elevated values at T2.

In agreement with previous studies, the harvest date had a significant effect on lactone content in all genotypes [26]. The content of *γ*-dodecalactone, one of those impactful volatiles, varied significantly among the different harvest dates in both two- and four-time-point analyses; the highest concentration value was observed at T2, while for most genotypes, the lowest was determined later (T3). Butyrolactone had maximum values in T1 in four of the six genotypes. However, *γ*-decalactone, a known key aroma component, highly affected by environmental variations [37], showed statistically significant changes only at the two-time-points analysis.

Phenylpropanoids also showed statistically significant differences over the harvesting period. This is mainly due to the variation observed in the content levels of *trans*-cinnamic acid, which generally declined for most cultivars in spring harvests (T3 and T4) (Figure 2). A similar pattern was observed for the aromatic compounds and citraconic anhydride (their concentration also decreased in spring) (Figure 2). Furanones, instead, were not significantly affected by harvest date, with all genotypes showing almost similar high and constant content levels of mesifurane and furaneol throughout the seasons, except G8. The latter stood out among the rest for its much greater content, which was especially higher in T1. No statistically significant impact of harvest time on short-chain acid content was observed; however, higher levels were detected at T2 for all genotypes, except for ‘Victory’, which exhibited the highest concentration at T1 (Figure S1).

In general, the observation that the highest content of certain aroma-impactful volatiles was detected at T1 and T2, while a significant reduction occurred near the end of the harvesting period, implies that fruit that is harvested later, at the same ripening stage though, contains less volatiles which eventually deprives the fruit’s overall liking [27]. Presumably, this is associated with crop load, which in strawberries, as in many other plants, can affect the quality of the fruit [38,39]. At the beginning of the season, the fruit/leaves ratio is low; thus, photosynthetic assimilates are available for a few fruits, so there is a surplus of energy for secondary compounds. In contrast, later in the season, the ratio is high; therefore, the demand for assimilates is allocated to more fruits, thus affecting fruit quality by ‘sacrificing’ volatiles and other secondary compounds. Correia et al. [40] reported that crop load was negatively correlated with quality traits, such as titratable acidity, in strawberry cultivars; however, as they pointed out, this effect varied among the cultivars studied, indicating that the genotype is an important factor in determining fruit quality parameters.

The decrease in the volatile content may also be due to environmental factors (different temperatures, rainfall, daylight, and duration) or cultivation practices. In support of this hypothesis, earlier studies that examined extra-early or early fruit production until late production has shown that organic acids increase from January to May, whereas the phenolics show a bell-shaped distribution with a peak in March [41]. Other parameters that affect the volatile production include ethylene. Strawberry is classified as a non-climacteric fruit, yet it is known that ethylene affects strawberry ripening and plays a key role in the development of strawberry color, in the accumulation of taste-related compounds (flavonoids, phenolics, organic acids, and sugars), and in the softening process [42].

Our findings indicate that the genotype and the harvest time strongly affect the volatile composition of strawberries, with quantitative rather than qualitative differences observed among the genotypes studied. Regarding the commercial varieties, ‘Rociera’ was evidenced to be more sensitive to seasonal environmental factors with its more pronounced changes throughout the four harvest dates, showing a much higher content of lactones, aromatic compounds, esters, and short-chain acids than the rest of the genotypes, particularly when harvested at T2. On the contrary, ‘Victory’, ‘Leyre’, and ‘Inspire’ showed rather stable volatile content patterns over the season. Concerning the genotypes from the Berryplasma collection (G2 and G8), G2 presents lower volatile content in general, but it exhibits a constant trend, with less significant changes in its volatile composition from November to April, indicating a major effect of the plant’s genotype, which could be a very attractive trait for selection and breeding. G8, in contrast, presents sharper variations but reveals a remarkable VOC profile, richer than that of commercial varieties, with the highest furanone and terpene content of all genotypes in three of the four harvest time points. Further research on the effect of fruit harvesting season is ongoing via a comparative genomic and transcriptomic analysis of the key genes that are responsible for the production of the corresponding volatile organic compounds.

## 3. Materials and Methods

### 3.1. Plant Material

This present study was conducted during the 2021/2022 growing season at Berryplasma World Ltd., Varda Ilias, Greece. All strawberry genotypes were cultivated under the same agronomic conditions and collected at two different time points (25 genotypes) and at four time points (6 genotypes) during the harvest period (from November 2021 to April 2022) from an experimental field located in Varda, municipality of Ilia (38.03135954886935, 21.356497184407626). Healthy, uniform, and fully ripe berries of each genotype were thoroughly selected for examination at each time point. Harvested fruit samples were transported to the laboratory at a temperature of 4 °C and immediately stored at −20 °C until analysis. Eight of them are commercial cultivars: ‘Rociera’ was developed by Nuevos Materiales (FNM), Huelva, Spain and launched in 2017; ‘Fortuna’ by the University of Florida, Gainesville, FL, USA, and is widely cultivated in many areas around the world; ‘Victory’ and ‘Inspire’ by Plant Sciences/Berry Genetics in Interlaken, Santa Cruz, CA, USA; ‘Fronteras’ by the former UC plant breeders Doug Shaw and Kirk Larson, Davis, CA, USA and launched in 2014; ‘Leyre’ (A14 09 cv.) by Masiá Ciscar S.A., Huelva, Spain; ‘Savana’ and ‘Plared-1075’ by Planasa, Cartaya, Spain. The rest of genotypes have been developed by Berryplasma World Ltd, Varda, Greece. Three of them are new Berryplasma World Ltd. varieties (‘Elektra’/G13, ‘Kallisti’/G14, and ‘Phaedra’/G15), five of them are genotypes from the collection of Berryplasma World Ltd. (G10, G11, G12, G16, and G17), and nine of them are genotypes derived from an initial population of 30,000 seedlings of the advanced stage selection of Berryplasma World Ltd. breeding program (G1, G2, G3, G4, G5, G6, G7, G8, and G9). The experiment was conducted under commercial conditions in greenhouses measuring 8.2 × 3.5 × 60 m each (W × H × L). Mother plants of each variety were planted in 10 L pots in the summer of 2021 and grown until they produced runner tips. All runner tips from each genotype were harvested in July 2021 and placed in 66-place trays measuring 40 × 60 cm and holding 118 cc. All genotypes were propagated and cultivated under the same conditions until planting in plastic tunnels of the R&D department. The irrigation and fertilization schedule were the same for all strawberry plants and standard commercial nutrient solution (N: 150 ppm, P: 50 ppm, K: 180 ppm, Ca: 200 ppm, Mg: 40 ppm, Fe: 10 ppm, Mn: 0.32 ppm, Zn: 0.1 ppm, B: 0.2 ppm, Cu: 0.05 ppm, and Mo: 0.04 ppm, pH 6.00) was applied through a drip irrigation system. The nutrient solution was administered using a Dosatron dosing pump (Dosatron International, Bordeaux, France) with an electrical conductivity of 1.6 mS. Plug plants of all genotypes were planted in 1 M coir growbags (Dutch Plantin Coir India Pvt. Ltd., Coimbatore, India) and were placed in raised beds at a density of 5 plants per growbag and 74,000 plants per ha in total (7.4 plants/m^2^).

### 3.2. Fruit Measurements and Experimental Design

Strawberry fruit was harvested at 6-day intervals during the autumn/winter period (November–February) and at 2-day intervals throughout the spring period (until 30 April 2022). The experiment was conducted as a randomized complete block design with 25 genotypes replicated 4 times (*n* = 4). Each replication consisted of 120 plants. All harvested fruit (∼600–800 g) were used to investigate the volatile compounds of each genotype by GC-MS analysis. Samples for 25 genotypes and replication were collected at two time intervals—T1: late November–December (22 November–31 December), T3: February–early March (1 February–15 March), and samples for 6 genotypes and replication were collected at four time intervals—T1: late November–December (22 November–31 December), T2: January (1 January–31 January), T3: February-mid March (1 February–15 March) and T4: April (1 April–30 April). Each sample consisted of a minimum of 500 g of fruit per replication.

### 3.3. Reagents and Chemicals

Ethyl Acetate (EtOAc) and sodium chloride (NaCl), certified ACS reagents, ≥99.5%, used in all the experimental work were obtained from Fisher Scientific (Pittsburgh, PA, USA). Tetrahydrofuran (THF), HPLC grade was provided by ChemLab (Eernegem, Belgium). Saturated alkane standards C_8_–C_20_ and C_21_–C_40_ in hexane were provided by Sigma-Aldrich (St. Louis, MO, USA).

### 3.4. Preparation of Samples for Analysis of Strawberry Volatiles

The samples were prepared as described in Leonardou et al. [30], with minor modifications. Each sample (∼600–800 g) was homogenized in a blender with 30–40 mL of a NaCl-saturated solution. The resulting fruit puree was centrifuged at 13.000 × rpm for 30 min at 4 °C, and approximately 30 g of the supernatant was used to extract the strawberry volatile compounds. An equal amount of NaCl-saturated solution (~30 mL) was then added. Ethyl acetate (EtOAc) was subsequently added (60 mL), and the mixture was stirred at room temperature for 45 min. The layers were separated, and the aqueous phase was extracted with EtOAc (3 times, 30 mL/time). The combined organic phases were dried over Na_2_SO_4_ and concentrated in vacuo to a volume of ~1 mL. Then, the solution was transferred to a 2 mL vial, and the solvent was evaporated with a stream of nitrogen. The obtained extract was stored at −20 °C. For the determination of volatile components, the dry extract was redissolved in distilled tetrahydrofuran (HPLC grade).

### 3.5. Gas Chromatography–Mass Spectrometry (GC-MS)

All samples were analyzed using a 6890 N Network GC System equipped with a 5975B mass selective detector (MSD) from Agilent Technologies (Santa Clara, CA, USA) in the electron impact (EI) mode of 70eV. Profiling of VOCs was performed according to Ayala-Zavala et al. [29] with slight modifications. In brief, the capillary column was HP-5MS (30 m × 0.25 mm, 0.25 μm) with helium as carrier gas with flow 1 mL min^−1^, in a splitless mode, and the *m*/*z* range was 38–1050. The injection volume was 1 μL. The column oven temperature was held for 1 min at 40 °C and then raised at 4 °C min^−1^ until it reached 230 °C, and eventually ramped to 280 °C at 10 °C min^−1^ and held at the same temperature for 2 min. The method’s run time was 55 min.

### 3.6. Identification and Relative Quantification of Strawberry Volatiles

According to the total ion chromatogram of VOCs in strawberries, various mass spectral libraries (NIST08/NIH Mass Spectral Library/Robert Adams) were used as a starting point for identification. Elements with a similarity score greater than 800 were considered as candidate recognition, and the results were confirmed via comparison of their retention indices (RI) and EI mass spectra with those of reference compounds in the literature. The RI_exp_ values were calculated via the equation of Van den Dool and Kratz [43], using a series of saturated alkane standards C_8_–C_20_ and C_21_–C_40_ (Sigma-Aldrich, St. Louis, MO, USA), run under the same chromatographic conditions. For each volatile component, the peak area was calculated from the total ion chromatogram (TIC) with the program WSEARCH32 (Ver. 16/2005), and a normalization of values among cultivars according to the fresh sample concentration took place. The samples were analyzed in duplicates.

### 3.7. Statistical Analysis of Strawberry Volatiles

Statistical analysis was performed using IBM SPSS Statistics for Windows, version 28 (IBM Corp., Armonk, NY, USA). ANOVA and Linear Mixed Model (LMM) tests were used to statistically analyze the VOCs in relation to genotype and harvest time point, respectively, evaluating the impact of the factors studied on every variable. Significant differences were accepted if the *p*-value was ≤0.05. Boxplots T1/T3 were obtained from “ggplot2” package using R 2022, V4.2.2.

## 4. Conclusions

Key volatiles with an impact on aroma have been determined in various strawberry genotypes at different time points covering the whole harvest period. Our study demonstrates that in most genotypes, the concentration of certain important volatiles is reduced over time; however, changes in volatile content are not uniform. Based on our results, volatile emission depends on the harvest time but is also affected by the genotype. In that sense, screening of VOCs can be included in the evaluation of strawberry genotypes, enabling growers and breeders to identify those having specific quality characteristics related to the aroma. Moreover, developing genotypes with a highly volatile composition and a suitable, stable, and pleasing to people aroma and flavor maintained throughout the season is important for breeding programs since cultivars with these traits meet the overall consumers’ preferences and satisfy the food industry needs.

## Figures and Tables

**Table 1:** List of main VOCs identified in 25 cultivars of strawberry fruit at harvest points T1 and T3. Most significant volatiles according to [6] are highlighted in bold. The experimental and literature retention indices (RIs) are presented along with the compound category and the range of peak area percentages (%). The impact of the genotype (F-value) on the variability of the volatiles is shown in the last column.

Compound Name	Compound Category	RI_exp_	Range of Peak Area Percentages (%) ^a^ (T1)	Range of Peak Area Percentages (%) ^a^ (T3)	Genotype (F-Values) ^b^
2-Methylpropanoic acid	Acids	777	0.29–1.36	0.10–1.83	1.742
**Ethyl butanoate**	Esters	804	0.38–2.48	0.32–0.99	0.732
**Butanoic acid**	Acids	808	0.71–6.08	0.17–4.27	2.989 **
**Ethyl 2-methylbutanoate**	Esters	849	0.12–0.86	0.06–0.20 ^d^	0.641
Maleic anhydride ^c^	Anhydrides	862	0.86–6.12	≤1.22 ^e,f^	0.777
**2-Methylbutanoic acid**	Acids	871	0.27–4.59	0.48–9.63	1.681
Butyrolactone	Lactones	916	0.17–7.83	0.18–6.72	0.771
**Methyl hexanoate**	Esters	926	0.24–2.54	0.34–4.93	1.053
Citraconic anhydride ^c^	Anhydrides	948	0.85–2.93	≤2.23 ^e,f^	0.886
**Ethyl hexanoate**	Esters	1001	0.46–3.42	0.66–6.07	1.771
**Hexanoic acid**	Acids	1009	0.73–31.88	1.31–36.56	1.509
Succinic anhydride ^c^	Anhydrides	1027	0.34–5.38	≤1.90 ^e,f^	0.834
Limonene	Terpenes	1028	0.17–1.01	0.09–0.97	1.468
Benzyl Alcohol	Aromatic compounds	1038	0.31–1.89	0.40–2.96	1.248
**Mesifurane**	Furanones	1063	0.09–9.92	0.37–7.36	2.450 *
**Furaneol**	Furanones	1070	0.62–12.15	2.19–20.66	3.285 **
*trans*-Linalool oxide	Terpenes	1089	0.19–1.04	0.23–1.92	1.970 *
**Linalool**	Terpenes	1101	0.39–3.91	0.26–3.85	2.167 *
Coumaran	Phenylpropanoids	1227	1.30–5.07	1.82–7.22	1.316
*trans*-Cinnamic acid ^b^	Phenylpropanoids	1481	6.96–64.42	9.99–55.42	0.516
***γ*-Decalactone**	Lactones	1472	1.18–12.56	2.35–13.80	2.656 *
Levoglucosan	Others	1553	0.27–9.48	0.51–11.83	0.757
*trans*-(*Ε*)-Nerolidol	Terpenes	1567	0.53–4.60	0.18–2.60	0.913
Bisabolol oxide II	Terpenes	1659	1.08–6.01	1.45–5.15	0.617
***γ*-Dodecalactone**	Lactones	1682	0.22–0.97	0.13–1.08 ^e^	0.943
Hexadecanoic acid	Acids/Fatty acids	1968	0.79–9.85	0.34–12.52	0.637
Oleic and Elaidic acid	Acids/Fatty acids	2141	0.61–5.08	1.40–6.84	0.756
Stearic acid	Acids/Fatty acids	1682	0.22–0.97	0.38–3.19	0.754

^a^ Average %content of each of the compounds of all genotypes exceeds ≥0.3%. ^b^ F values are determined via ANOVA; asterisks indicate a statistically significant change in values based on genotypes (* 0.05 ≥ *p* > 0.01; ** 0.01 ≥ *p*> 0.001). ^c^ There are not strictly considered volatile substances. ^d^ Average % content of 0.05% for ethyl 2-methylbutanoate. ^e^ Average % content ≤ 0.3%. ^f^ The absence of a lower value in the Range of Peak Area Percentages (‘≤’ is used instead) indicates that >80% of the analyzed genotypes do not present the compound in question.

**Table 2:** Volatile components and categories with statistically significant change over two and/or four harvest time points. F values are presented on the left for the 25 genotypes examined at T1/T3 and on the right for the 6 genotypes examined at T1/T2/T3/T4 (G2, G8, ‘Rociera’, ‘Victory’, Leyre’, and ‘Inspire’).

Volatile Constituent	F-Value (Significance)	
	Harvest Date (Two-Point T1/T3)	Harvest Date (Four-Point T1/T2/T3/T4)
Esters	7.179 **	1.726
Terpenes	15.358 ***	2.151
Phenylpropanoids	16.972 ***	3.701 *
Aromatic compounds	0.245	6.019 **
Short Chain acids	5.756 *	2.433
Lactones	7.015 *	4.229 *
**Ethyl butanoate**	7.836 *	0.467
**Butanoic acid**	5.659 *	0.375
**Ethyl 2-methylbutanoate**	16.877 ***	3.983 *
Butyrolactone	10.797 **	3.857 *
Citraconic anhydride	3.034	6.511 *
**Ethyl hexanoate**	4.800 *	2.537
**Hexanoic acid**	5.561 *	2.573
Limonene	4.219 *	1.456
**Furaneol**	4.813 **	0.019
**Linalool**	9.530 **	2.114
*trans*-Cinnamic acid	19.073 ***	4.008 *
***γ*-Decalactone**	7.498 *	1.736
*trans*-(*Ε*)-Nerolidol	15.152 ***	4.307 *
Bisabolol oxide II	11.866 **	1.416
***γ*-Dodecalactone**	9.957 **	7.291 **

Asterisks indicate a statistically significant change in values based on harvest date (* 0.05 ≥ *p* > 0.01; ** 0.01 ≥ *p* > 0.001; *** *p* ≤ 0.001).

## Data Availability

Data is contained within the article or supplementary material.

## Supplementary Materials

The following supporting information can be downloaded at: https://www.mdpi.com/article/10.3390/plants12091881/s1, Table S1: List of 25 strawberry genotypes examined; Table S2: List of abundant identified VOCs (>0.1% at least at one time point) at T1 and T3 for the 25 genotypes examined; Table S3: Percentage content of the main categories of VOCs at the two different time points, T1 and T3, for the 25 genotypes; Table S4: Percentage content of the main categories of VOCs at the four different time points (T1, T2, T3, T4) for the six selected genotypes; Figure S1: Evolution of the content levels of volatile categories in the six selected genotypes over harvesting periods T1–T4.
