# Supplementary material for: Monitoring of Volatile Organic Compounds in Strawberry Genotypes over the Harvest Period
Source: Plants (Basel). 2023 May 4;12(9):1881. doi: 10.3390/plants12091881 (PMC10181119; doi:10.3390/plants12091881)

Table S1. List of 25 strawberry genotypes examined, which consist of 9 strawberry genotypes from the advanced selections of Berryplasma breeding program, 5 genotypes from the Berryplasma collection, 3 new varieties developed at Berryplasma and 8 basic varieties grown in the region as controls of foreign breeding programs.

Genotypes	Group
G1	Advanced
G2	Advanced
G3	Advanced
G4	Advanced
G5	Advanced
G6	Advanced
G7	Advanced
G8	Advanced
G9	Advanced
G10	Collection
G11	Collection
G12	Collection
G13	Berry_CV
G14	Berry_CV
G15	Berry_CV
G16	Collection
G17	Collection
G18	Control/Rociera
G19	Control/Victory
G20	Control/Fortuna
G21	Control/Fronteras
G22	Control/Leyre
G23	Control/Inspire
G24	Control/Savana
G25	Control/ Plared-1075

Table S2. List of abundant identified VOCs (>0.1% at least at one time point) at T1 and T3 for the 25 genotypes examined. The experimental and literature retention indices (RIs) are presented along with the compound category, the range of % peak area percentages, as well as references to whether it affects flavor and/or references to previous occurrence in strawberries.

No	RI_{exp}	RI_{lit}	Compound	Compound Category	Flavour impact	Range of Peak Area Percentages
1	760	712	4-Methyl-2-pentanone	Ketones		<1.25
2	772	743	Ethyl 2-methylpropanoate	Esters	a(sweet, apple fruity	0.08-0.52
3	777	785	2-Methylpropanoic acid (Isobutyric acid)	Acids	a(rancid, butter, cheese)	0.29-1.36
4	783	753	2-Methylpropyl acetate (Isobutyl acetate)	Esters	a(fruit, apple, banana)	0.10-0.27
5	804	802	Ethyl butanoate	Esters	a(apple), b(estery, fruity, sweet)	0.38-2.48
6	808	790	*n*-Butanoic acid	Acids	a(rancid, cheese, sweat), b(cheesy, fruity)	0.71-6.08
7	815	807	Butyl acetate	Esters	a(pear)	0.12-0.67
8	832	811b	Methyl 2-hydroxybutanoate	Esters		0.08-0.56
9	849	843	Ethyl 2-methylbutanoate	Esters	ifra (fruity,strawberry,peach)	0.12-0.86
10	852	832	Ethyl 3-methylbutanoate (coeluted with an unidentified compound)	Esters	a (fruit)	<0.47
11	858	826	3-Methylbutanoic acid (Isovaleric acid)	Acids	a (sweat,acid,rancid) ifra(acidic,cheesy,fruity)	<2.03

12	860	830	Methyl 3-hydroxybutanoate	Esters		<0.19
13	862	827	Maleic anhydride (cis-butenedioic anhydride; 2,5-furandione)	Anhydrides		0.86-6.12
14	871	846/886	2-Methylbutanoic acid	Acids	b(cheesy, stinky),g	0.27-4.59
15	903	901	Ethyl pentanoate	Esters	a(yeast,fruit)	<0.64
16	916	915/916	Butyrolactone [dihydrofuran-2(3H)-one]	Lactones	a(caramel-sweet)	0.17-7.83
17	926	924	Methyl *n*-hexanoate (methyl caproate)	Esters	a(fruit, fresh, sweet)	0.24-2.54
18	948	949	Citraconic anhydride (3-methyl-2,5-furandione)	Anhydrides		0.85-2.93
19	961	927	Benzaldehyde	Aromatic compounds	a(almond, burnt sugar)	0.15-2.79
20	976	977	3-Hydroxybutanoic acid	Acids		<4.26
21	1001	996	Ethyl hexanoate (ethyl caproate)	Esters	a(apple, peel, fruit), b (fruity, sweet)	0.46-3.42
22	1009	977/981	*n*-Hexanoic acid (*n*-caproic acid)	Acids	b(sour, cheesy)	0.73-31.88
23	1016	998	4-Carene	Terpenes		<0.74
24	1016	1007	Hexyl acetate	Esters	a(fruit, herb) ifra(fruity,green,apple)	0.12-0.64
25	1019	997	(*E*)-2-Hexenyl acetate	Esters	ifra(green, sweet, fruity)	0.28-0.98
26	1027	1022(non-polar)	Succinic anhydride (dihydro-2,5-furandione)	Anhydrides		0.34-5.38
27	1028	1007/1020/1022/1025-1033	Limonene	Terpenes	a(citrus, mint)	0.17-1.01

28	1038	1020/1033-1036/1042	Benzyl Alcohol	Aromatic compounds	a(sweet, flower),g	0.31-1.89
29	1039	1036	(*E*)-β-ocimene	Terpenes	a(sweet,herb)	<0.17
30	1040	967(DB-1, Normal alkane)	Itaconic anhydride (Dihydro-3-methylene-2,5-furandione)	Anhydrides		<2.63
31	1040	1014b	Pantolactone [dihydro-3-hydroxy-4,4-dimethyl-2(3H)-furanone ; 2-hydroxy-3,3-dimethyl-*γ*-butyrolactone]	Lactones	a(cotton candy)	not measurable (coeluting peak with itaconic anhydride)
32	1049	1005	3-carene	Terpenes	ifra(herbal, green, pine)	<0.97
33	1057	1056	*γ*-Hexalactone (*γ*-ethyl-*γ*-butyrolactone, dihydro-5-ethyl-2(3H)-furanone, *γ*-Caprolactone)	Lactones	ifra(fruit,coconut,herbal)	<0.35
34	1063	1065	Mesifurane [2,5-dimethyl-4-methoxy-3(2H)-furanone; DMMF]	Furanones	b(toffee, sugary, sweet),g	0.09-9.92
35	1070	1055/1072/1097	Furaneol [2,5-dimethyl-4-hydroxy-3(2H)-furanone; DMHF]	Furanones	a(caramel),b(sweet, candy, caramellic), g	0.62-12.15
36	1089	1065/1069/1087-1088/1091	*trans*-Linalool oxide (furanoid) (trans-5-ethenyltetrahydro-	Terpenes	a(flower)	0.19-1.04

			α,α,5-trimethyl-2-furanmethanol)			
37	1098	1084 (DB-5)	δ-Hexalactone [tetrahydro-6-methyl-2H-pyran-2-one; δ-caprolactone]	Lactones	ifra(fruity,coconut,creamy)	<0.26
38	1101	1079/1082/1092/1094/1097-1105	Linalool (3,7-dimethyl-1,6-octadien-3-ol)	Terpenes	a(flower, lavender),b(floral)	0.39-3.91
39	1116	-	Levoglucosenone	Others		0.17-0.36
40	1131	1161	2-Ethyl hexanoic acid	Acids		<0.34
41	1173	1163 (DB-5MS)	4-Ethylphenol	Aromatic compounds	a(must)	0.32-0.62
42	1183	1159/1178	Benzoic Acid	Aromatic compounds	a(urine) ifra(herbal,balsamic,powdery	0.58-2.28
43	1191	1187/1192	1-Dodecene	Alkenes	a(alkane)	0.40-1.41
44	1210	1188	*p*-Menth-1-en-9-al	Terpenes		0.18-0.86
45	1227	1223-1224(SPB-5)	Coumaran (2,3-dihydrobenzofuran)	Phenylpropanoids	e(green tea)	1.30-5.07
46	1362	1323	Eugenol	Phenylpropanoids	ifra(spicy,clove,floral)	<1.25
47	1481	1450/1462 (DB-1)	*trans*-Cinnamic acid [(*E*)-3-phenyl-2-propenoic acid]	Phenylpropanoids	a(honey)	6.96-64.42
48	1472	1470	γ-Decalactone (5-hexyldihydro-2(3H)-furanone)	Lactones	a(peach, fat), b(sweet, peach, lactonic)	1.18-12.56

49	1553	1491	Levoglucosan (1,6-anhydro-β-D-glucopyranose)	Others		0.27-9.48
50	1567	1544/1561-1563/1568/1569	*trans*-(*E*)-Nerolidol [(*E*)-3,7,11-trimethyldodeca-1,6,10-trien-3-ol]	Terpenes	a(wax)	0.53-4.60
51	1571	1559/1566/-1568/1570/1573/1576	*n*-Dodecanoic acid (lauric acid)	Fatty acids	a(metal)	<0.70
52	1659	1658	Bisabolol oxide II	Terpenes		1.08-6.01
53	1682	1675	*γ*-Dodecalactone [Dihydro-5-octyl-2(3H)-furanone]	Lactones	a(sweet, flower, fruit),g	0.22-0.97
54	1968	1962/1963/1969/1971/1972/1975/1977/1978/1991/1995/2003	*n*-Hexadecanoic acid (palmitic acid)	Fatty acids		0.79-9.85
55	2135	2095/2104/2130/2140/2144/2170	Linoleic acid [(9Z,12*Z*)-octadeca-9,12-dienoic acid]	Fatty acids		0.22-1.15
56	2141	2102/2141/2152/2175	Oleic Acid [(*Z*)-octadec-9-enoic acid]	Fatty acids	a(fat)	not measurable (coeluting peak with elaidic acid)
57	2141	2141	Elaidic acid, *trans*-(*E*)-oleic acid	Fatty acids		0.61-5.08
58	2166	2172/2178/2180/2188	Stearic acid (Octadecanoic acid)	Fatty acids		0.32-5.82

The absence of a lower value in the Range of Peak Area Percentages (instead '<' is used) indicates that >80% of the analyzed genotypes do not present the compound in question. (For references, see our previous work in Leonardou et al. [30].

Table S3. Percentage content of the main categories of VOCs at the two different time points, T1 and T3, for the 25 genotypes. Asterisks indicate a statistically significant change in values between the two time points ($p<0.001$).

G	Group	Esters		Terpenes		Phenylpropanoids		Aromatics		Short chain acids		Fatty acids		Furanones		Lactones		Anhydrides	
		**T1**	**T3**	**T1**	**T3**	**T1**	**T3**	**T1**	**T3**	**T1**	**T3**	**T1**	**T3**	**T1**	T3	**T1**	**T3**	**T1**	**T3**
G1	1stPriority	5.44	6.33	4.14	5.20	59.52	12.48	0.00	3.60	13.16	14.50	1.24	19.01	1.12	3.79	3.63	0.22	0.54	0,00
G2	1stPriority	4.26	1.38	3.29	3.31	41.90	58.64	2.99	1.64	7.89	7.77	0.85	4.63	1.39	3.78	3.02	0.68	0.37	3,49
G3	1stPriority	5.14	1.75	7.07	8.11	44.80	33.89	0.56	4.10	13.75	22.56	5.36	0.00	0.38	3.85	7.56	10.47	1.66	0,00
G4	1stPriority	3.08	2.20	3.42	2.33	64.42	57.38	0.22	3.90	8.33	8.67	0.00	1.24	0.72	2.85	3.91	0.47	6.64	3,45
G5	1stPriority	7.11	4.75	7.50	7.89	33.78	28.58	1.95	3.53	29.94	15.70	0.00	0.00	7.37	8.93	10.65	2.09	0.54	0,00
G6	1stPriority	2.61	2.31	14.49	5.84	31.05	23.37	0.00	2.71	11.80	11.65	0.00	15.86	6.03	15.51	2.37	1.36	0.00	1,41
G7	1stPriority	2.77	5.17	4.76	6.52	61.35	35.01	2.57	7.28	18.35	9.57	1.59	1.09	2.50	12.97	2.41	2.18	2.29	0,00
G8	1stPriority	4.06	4.80	7.96	8.45	6.96	0.00	0.00	0.00	5.02	4.99	1.70	3.18	19.62	24.03	5.48	3.64	1.59	0,00
G9	1stPriority	5.27	2.01	7.41	2.74	23.31	27.51	0.40	0.83	19.06	6.39	0.00	12.51	18.24	15.60	0.68	1.90	0.34	0,97
G10	PARENT	2.56	1.94	4.91	9.34	32.81	28.26	0.00	0.00	2.88	4.13	2.59	0.00	0.36	0.00	1.32	6.72	3.01	0,00
G11	PARENT	3.39	9.61	6.50	5.10	25.22	10.72	2.51	5.47	15.64	34.83	17.80	0.00	1.24	12.97	9.61	10.79	<0.01	0,00
G12	PARENT	1.24	2.68	2.88	4.02	22.78	9.99	0.00	2.08	12.64	18.56	2.35	7.90	5.52	13.47	1.84	2.12	15.82	0,00
G13	Berry_CV	4.90	7.81	14.14	13.17	54.58	33.22	0.00	1.49	5.76	6.70	0.00	0.00	0.09	12.28	7.18	5.52	0.00	0,00
G14	Berry_CV	7.27	5.68	4.82	3.29	49.17	42.62	0.00	5.95	25.37	11.02	0.00	0.00	2.46	15.90	4.83	6.46	0.00	0,00
G15	Berry_CV	4.30	10.75	6.07	8.02	33.32	12.61	1.53	0.00	36.31	37.67	9.20	0.00	0.71	9.55	8.56	6.35	0.00	0,00
G16	COLLECTION	5.10	0.57	9.54	6.40	47.22	30.16	0.23	1.95	18.09	9.98	1.30	2.87	0.80	0.37	5.86	0.00	0.00	0,00
G17	COLLECTION	6.40	3.42	7.90	5.95	40.15	21.03	0.00	0.83	7.97	8.22	1.15	0.85	2.66	3.18	12.06	10.64	<0.01	0,00
G18	'Rociera'	4.08	8.15	3.82	4.00	24.69	16.90	0.71	1.16	13.55	47.13	0.32	2.04	2.90	5.99	7.11	7.57	6.35	0,00
G19	'Victory'	2.06	2.80	3.93	4.03	51.98	37.70	2.36	2.95	17.61	18.68	3.01	0.00	7.30	14.35	2.75	7.34	7.03	0,00
G20	'Fortuna'	2.51	8.99	7.68	10.13	43.31	21.98	1.59	5.22	17.98	15.80	0.00	0.00	0.46	4.92	13.68	14.63	<0.01	0,00
G21	'Fronteras'	2.28	4.07	7.34	4.38	54.85	45.37	1.07	2.22	5.86	8.17	17.58	4.93	5.80	8.56	5.09	7.51	0.00	0,00
G22	'Leyre'	5.43	2.44	9.21	3.48	19.95	15.78	0.00	0.40	38.90	18.09	0.00	0.34	8.17	18.44	17.85	13.74	0.00	0,00
G23	'Inspire'	4.81	7.61	3.47	8.36	46.92	29.52	0.00	4.48	17.34	9.66	0.00	0.00	3.52	16.80	3.75	5.30	0.00	0,00
G24	'Savanna'	4.29	7.64	4.98	7.57	29.63	12.36	1.22	3.01	23.85	11.43	7.33	0.00	3.06	13.49	13.04	15.70	0.00	0,00
G25	'Plared-1075'	3.04	3.64	4.77	4.59	33.31	18.66	4.31	7.18	24.16	46.09	1.70	0.00	0.00	1.49	13.16	16.69	<0.01	0,00
Average ± Standard Deviation		4.14 ±1.54	4.74 ±2.84	6.48 ±2.98	6.09 ±2.60	**39.08 ±14.28**	**26.55 ±14.24***	**0.97 ±1.18**	**2.88 ±2.13***	16.45 ±9.18	16.32 ±12.04	3.00 ±4.91	3.06 ±5.18	**4.10 ±5.02**	**9.72 ±6.32***	6.70 ±4.48	6.40 ±5.01	1.85 ±3.57	0.37 ±0.97

Table S4. Percentage content of the main categories of VOCs at the four different time points (T1, T2, T3, T4) for the 6 selected genotypes.

Entry	G2				G8				'Rociera'				'Victory'				'Leyre'				'Inspire'			
	T1	T2	T3	T4	T1	T2	T3	T4	T1	T2	T3	T4	T1	T2	T3	T4	T1	T2	T3	T4	T1	T2	T3	T4
**Esters**	4.26	4.19	1.38	2.23	4.06	1.55	4.80	4.52	4.08	3.20	8.15	4.64	2.06	0.59	2.80	3.68	5.43	3.40	2.44	5.12	4.81	5.02	7.61	4.97
**Terpenes**	3.29	3.83	3.31	2.39	7.96	6.11	8.45	7.10	3.82	2.95	4.00	3.25	3.93	2.00	4.03	2.31	9.21	3.05	3.48	5.39	3.47	5.47	8.36	6.09
**Phenypropanoids**	41.90	61.53	58.64	30.80	6.96	11.69	0.00	2.41	24.69	38.68	16.90	22.32	54.39	54.99	37.70	37.86	19.95	7.88	15.78	18.10	44.92	45.19	29.52	23.51
**Aromatics**	2.99	4.39	1.64	2.88	0.00	0.34	0.00	3.37	0.71	2.68	1.16	1.69	2.36	2.38	2.95	2.44	0.00	1.79	0.40	5.94	0.00	3.54	4.48	5.68
**Short chain acids**	7.89	15.31	7.77	2.43	5.02	15.45	4.99	14.87	13.55	26.90	47.13	24.41	17.61	16.21	18.68	7.41	38.90	28.29	18.09	21.68	17.34	22.32	9.66	15.97
**Fatty acids**	0.85	2.78	4.63	1.62	1.70	5.50	3.18	0.65	0.32	3.78	2.04	17.50	3.01	2.55	0.00	15.12	0.00	15.69	0.34	0.00	0.00	0.34	0.00	2.38
**Furanones**	1.39	4.03	3.78	2.67	19.62	12.85	24.03	28.89	2.90	1.79	5.99	3.87	7.30	5.10	14.35	3.87	8.17	6.30	18.44	7.92	3.52	10.03	16.80	9.15
**Lactones**	3.02	2.14	0.68	1.78	5.48	7.58	3.64	8.64	7.11	17.28	7.57	4.81	2.75	9.82	7.34	2.71	17.85	7.48	13.74	10.37	3.75	6.09	5.30	7.89
**Anhydrides**	0.37	1.29	3.49	5.36	1.59	0.76	0.00	1.42	6.35	0.52	0.00	1.25	7.03	3.73	0.00	1.84	0.00	0.00	0.00	1.91	0.00	2.00	0.00	3.21
2-Methylpropanoic acid	0.00	0.00	0.00	0.00	0.00	0.38	0.00	0.47	0.29	0.41	0.69	0.52	0.61	0.18	0.00	0.23	0.63	0.53	0.76	0.84	0.69	0.40	0.59	0.65
Ethyl butanoate	0.95	0.84	0.32	0.69	0.64	0.43	1.27	0.90	0.77	1.15	2.52	1.39	0.70	0.47	1.14	0.50	1.80	1.29	1.61	2.26	1.08	0.90	1.77	1.30
Butanoic acid	3.57	0.00	0.00	0.00	0.71	2.62	0.00	1.80	6.07	6.44	10.65	3.86	0.85	0.00	0.85	0.62	4.85	8.15	4.27	7.20	1.08	2.89	1.21	2.18
Ethyl 2-methylbutanoate	0.00	0.06	0.00	0.06	0.00	0.00	0.10	0.00	0.14	0.00	0.00	0.07	0.30	0.00	0.00	0.06	0.78	0.06	0.07	0.19	0.46	0.00	0.12	0.07
Maleic anhydride	0.00	0.00	0.80	0.00	0.00	0.00	0.00	0.00	3.43	0.00	0.00	0.00	0.00	0.00	0.00	0.00	0.00	0.00	0.00	0.00	0.00	0.00	0.00	0.00
2-Methylbutanoic acid	2.87	2.13	1.14	0.70	0.00	0.19	0.93	1.12	0.27	3.55	3.59	1.70	1.17	0.00	0.94	0.12	1.54	1.20	1.67	0.99	0.99	3.24	1.93	1.95
Butyrolactone	2.48	0.68	0.18	0.93	0.00	0.66	0.79	1.54	0.90	0.67	2.55	0.91	2.35	0.56	3.65	2.39	5.29	0.48	4.64	2.18	3.47	0.29	2.53	1.67
Methyl hexanoate	0.83	0.71	0.51	0.51	0.00	0.28	0.91	0.68	0.24	0.21	0.55	0.54	0.31	0.00	0.87	0.39	1.94	0.41	0.00	1.13	1.69	0.35	3.14	0.65
Citraconic anhydride	0.00	0.00	0.79	1.81	1.59	0.76	0.00	1.00	0.85	0.19	0.00	0.53	1.65	0.95	0.00	0.57	0.00	0.00	0.00	1.28	0.00	0.54	0.00	1.32
Ethyl hexanoate	1.27	1.74	0.00	0.24	3.42	0.75	2.01	1.81	2.93	1.55	4.47	2.09	0.00	0.00	0.00	1.36	0.00	1.37	0.00	0.15	0.00	1.69	1.90	1.49
Hexanoic acid	1.11	13.18	6.64	1.73	3.18	12.26	4.07	11.43	6.92	14.29	32.20	17.34	10.72	12.02	16.33	5.13	31.88	15.61	11.39	11.17	14.59	15.78	5.93	9.99
Succinic anhydride	0.37	1.29	1.90	3.54	0.00	0.00	0.00	0.41	1.45	0.32	0.00	0.71	5.38	2.77	0.00	1.26	0.00	0.00	0.00	0.62	0.00	1.46	0.00	1.89
Limonene	0.17	0.20	0.12	0.18	0.98	0.52	0.61	0.33	0.31	0.00	0.13	0.00	0.00	0.28	0.00	0.00	1.01	0.24	0.40	0.28	0.38	0.00	0.28	0.00
Benzyl Alcohol	0.71	0.00	0.00	0.00	0.00	0.34	0.00	0.37	0.71	0.00	1.16	0.42	1.89	0.37	2.95	0.87	0.00	1.00	0.40	0.21	0.00	1.28	0.00	1.38
Mesifurane	0.77	2.02	1.23	1.36	7.47	7.15	3.37	17.26	1.08	1.46	1.64	1.42	3.11	1.90	3.50	1.11	3.58	3.30	6.34	7.02	1.59	2.72	4.43	3.41
Furaneol	0.63	2.01	2.55	1.31	12.15	5.70	20.66	11.63	1.81	0.34	4.35	2.45	4.19	3.20	10.85	2.76	4.59	2.99	12.12	0.90	1.93	7.31	12.37	5.74
trans-Linalool oxide	0.30	0.29	0.23	0.30	0.40	0.29	0.88	0.61	0.25	0.22	0.48	0.33	0.58	0.12	1.15	0.30	0.00	0.00	0.00	0.00	0.75	0.28	1.47	0.63
Linalool	0.89	0.37	0.33	0.32	1.55	0.90	2.50	1.56	0.58	0.46	0.85	0.55	0.48	0.00	0.00	0.21	2.57	0.71	<0.01	1.12	0.82	0.26	0.91	0.51
Coumaran	1.30	1.98	4.89	1.02	0.00	0.60	0.00	1.13	0.00	1.39	0.00	0.98	5.07	6.29	0.00	1.99	0.00	0.00	2.75	5.68	0.00	4.36	3.69	3.59
trans-Cinnamic acid	39.75	59.55	53.74	29.78	6.96	11.09	0.00	1.28	24.69	37.29	16.90	21.33	49.32	48.70	37.70	35.87	19.95	7.88	13.03	12.42	44.92	45.19	25.83	19.92

γ-Decalactone	<0.01	0.53	<0.01	0.64	5.03	6.27	2.86	6.65	5.31	15.42	5.02	3.03	0.00	8.67	3.69	<0.01	12.56	6.49	9.10	7.65	0.00	4.83	2.77	5.07
Levoglucosan	0.73	9.10	11.83	8.17	0.00	0.00	0.00	0.00	0.00	3.90	0.00	0.86	9.25	10.03	0.00	7.10	0.00	0.00	0.00	3.06	0.00	6.37	0.00	3.83
trans-(*E*)-Nerolidol	0.87	0.79	0.18	0.03	1.61	1.35	1.45	1.26	0.70	0.83	0.96	0.64	0.56	0.00	0.00	0.15	1.83	0.62	<0.01	0.89	1.53	0.88	0.60	1.19
Bisabolol oxide II	1.08	2.18	2.45	1.56	3.05	1.75	2.87	2.80	1.98	1.44	1.58	1.74	2.31	1.60	2.87	1.66	3.81	1.48	2.63	2.55	0.00	4.06	5.10	3.77
γ-Dodecalactone	0.54	0.68	0.50	0.20	0.45	0.65	<0.01	0.37	0.36	0.54	<0.01	0.45	0.40	0.33	0.00	0.31	0.00	0.50	0.00	0.36	0.28	0.73	0.00	0.77
Hexadecanoic acid	0.00	1.35	1.91	1.62	1.16	2.59	3.18	0.65	0.00	1.54	2.04	11.10	2.16	1.68	0.00	4.99	0.00	6.72	0.34	0.00	0.00	0.34	0.00	1.99
Oleic and Elaidic acid	0.00	0.77	1.61	0.00	0.00	1.59	0.00	0.00	0.00	1.30	0.00	3.73	0.00	0.00	0.00	6.08	0.00	5.41	0.00	0.00	0.00	0.00	0.00	0.00
Stearic acid	0.85	0.54	0.89	0.00	0.55	0.80	0.00	0.00	0.32	0.57	0.00	2.66	0.85	0.21	0.00	2.13	0.00	2.15	0.00	0.00	0.00	0.00	0.00	0.39

Figure S1. Evolution of the content levels of volatile categories in the 6 selected genotypes over harvesting periods T1-T4 (chromatographic peak area *vs.* harvest time point): (**a**) esters, (**b**) terpenes, (**c**) short chain acids, (**d**) fatty acids, (**e**) furanones, (**f**) anhydrides.

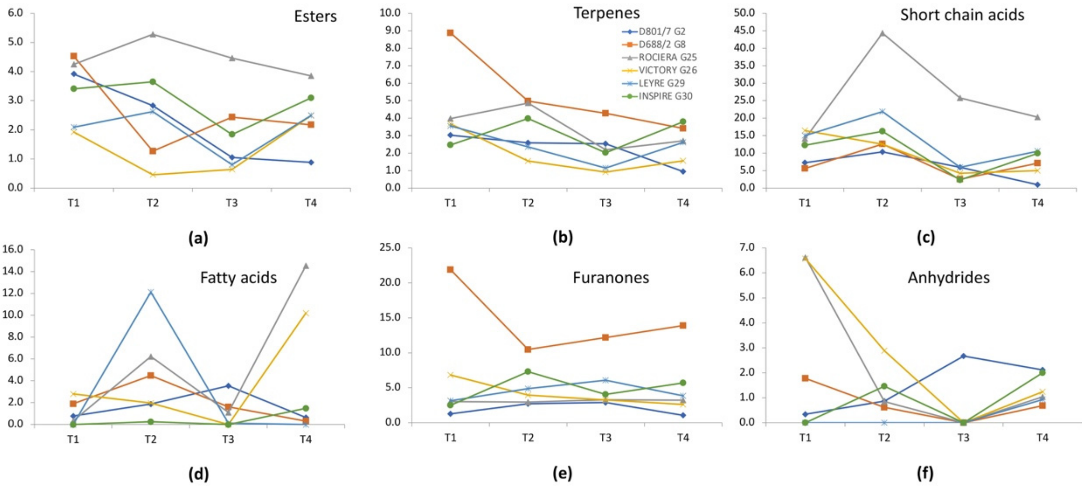

Supplement: Supplementary file 1 [file plants-12-01881-s001.zip › plants-2345593-supplementary.pdf]
